# Endocrine-disrupting pesticide exposure relevant to reproductive health: a case study from Costa Rica

**DOI:** 10.1007/s10661-025-14011-8

**Published:** 2025-04-16

**Authors:** Navilla Apú, François Rommes, Maricruz Alvarado-Arias, Michael Méndez-Rivera, Verónica Lizano-Fallas

**Affiliations:** 1 Instituto de Investigaciones Farmacéuticas (INIFAR), Facultad de Farmacia, Universidad de Costa Rica, San José, 2060 Costa Rica; 2 Département Agronomique, Haute Ėcole Charlemagne Huy, Huy, 4500 Belgium; 3 Centro de Investigación en Contaminación Ambiental (CICA), Universidad de Costa Rica, San José, 2060 Costa Rica

**Keywords:** Endocrine disruptors, Pesticides, Human reproductive health, Human biomonitoring, Risk assessment, Pesticide prioritization

## Abstract

Global pesticide use has increased significantly over the past decade, leading to greater exposure to contaminants and associated health risks. Endocrine-disrupting pesticides have gained attention due to their strong association with human reproductive impairments and rising global infertility rates. In Costa Rica, studies have reported reduced fertility among agricultural workers and a higher prevalence of male infertility in regions with intensive pesticide use. However, the prioritization of pesticides detected in human fluids, based on their potential impact on reproductive health, has not been conducted. Here, analyzing human biomonitoring studies in the country over the last 25 years, 13 pesticides were identified and prioritized. Mancozeb ranked highest (14.8%), followed by dieldrin (12.1%) and chlorothalonil (12.0%). Eight criteria were used for prioritization, with non-carcinogenic risk, reported reproductive effects, and endocrine disruptor classification as key factors. This comprehensive approach highlights how multiple criteria collectively inform pesticide prioritization in relation to reproductive health risks. The findings indicated that while Costa Rica is a regional leader in pesticide biomonitoring, significant gaps remain, including limited data on unstudied pesticides and general population exposures. Establishing robust biomonitoring programs and public health surveillance systems to generate updated data and support evidence-based prevention policies is recommended. Additionally, the results of this study provide a valuable framework for guiding future research on the potential effects of pesticide mixtures.

**Supplementary Information:**

The online version contains supplementary material available at 10.1007/s10661-025-14011-8.

## Introduction

The global use of pesticides plays a critical role in agriculture, as they are chemical compounds specifically designed to control and reduce pests (Zhou et al., 2025). Over the past decades, pesticide use in agriculture has significantly increased, reflecting both the growth of agricultural production and the intensification of farming systems. According to the Food and Agriculture Organization of the United Nations (FAO), global pesticide use reached 3.70 million tons in 2022, representing a 4% increase from 2021 and a 13% rise over the last decade (FAO, 2024). In Costa Rica, pesticide use is extensive, averaging 17.46 kg/ha in 2022 (FAO, 2024), especially in the production of export crops such as bananas and pineapple (Brühl et al., 2023). Unfortunately, along with the economic benefits of pesticide use, adverse health consequences arise due to their toxicity to non-target species, including humans. Pesticides contaminate our food, water, air, dust, and soil and can enter our bodies through ingestion, inhalation, and skin absorption (Segal & Giudice, 2019; Vessa et al., 2022). Moreover, pesticide exposure represents only a portion of our exposome, which refers to all the exposures an individual encounters over their lifetime that influence their health (Wild, 2005, 2012).

In the last years, global infertility rates have been on the rise (Skakkebæk et al., 2022), and associations between chemical exposure and reproductive impairments have been documented (Marlatt et al., 2022). Consequently, endocrine-disrupting (ED) pesticides have received increased attention (Ghosh et al., 2022). A pesticide is classified as an ED compound if there is evidence that it interferes with the endocrine system, resulting in harmful effects on an organisms’ health, development, or reproduction (Ewence et al., 2015). Some of the most recognized ED pesticides due to their adverse effects on reproduction are dichlorodiphenyltrichloroethane (DDT), mancozeb, malathion, dieldrin, and glyphosate (Bretveld et al., 2006; Moreira et al., 2021). Some of these substances, such as DDT, have been banned in the European Union and the USA due to their significant environmental impacts and potential risks to human health (PAN, 2022). In Costa Rica, pesticides like DDT and dieldrin have also been banned to minimize their impact on public health and ecosystems (SFE, 2024a). However, other ED pesticides, like glyphosate, remain widely used despite ongoing debates about their safety and regulatory status (SFE, 2024a).

The main negative effects associated with ED pesticide exposure reported for female reproduction health include reduced fertility, elevated risk of early pregnancy loss, and ovarian disfunction (Vessa et al., 2022). In men, exposure has been linked to decreased semen quality and testicular disfunction (Giulioni et al., 2022; Sharma et al., 2020). In Costa Rica, studies have reported lower fertility rates among agricultural workers exposed to pesticides and a higher prevalence of male infertility in regions with intensive pesticide use (Bustos-Obregón, 2001; Thrupp, 1991).

In this context, and within the framework of the exposome, the European RiskMix project identified a mixture of ED pesticides to which humans may be exposed, using existing data from various cohorts that investigated internal exposure profiles in populations of special concern, including developing children (Engelhardt et al., 2022). However, in Costa Rica, this type of mixture has not yet been identified, despite the availability of epidemiological studies from which this information could potentially be retrieved. Prioritizing pesticides from a list of exposure for further analyses involves a critical decision-making process. Complex problems of this type, which include multiple criteria, can be addressed mathematically by applying a multi-criteria decision analysis (MCDA) method. MCDA techniques offer foundational tools and approaches for identifying compromise solutions and therefore have broad applicability (Ishizaka & Nemery, 2013). In environmental toxicology, for example, these techniques have been used to rank chemicals for toxicological impact assessments (Gregoris et al., 2018; Tobiszewski et al., 2017), prioritize different dust sources (Kim et al., 2020), evaluate the risk of multi-ingredient dietary supplements (Oketch-Rabah et al., 2021), integrate protein targets of contaminants into the adverse outcome pathway framework (Lizano-Fallas et al., 2023), and predict cellular function disturbances caused by chemical mixtures (Lizano-Fallas et al., 2024).

In this study, we propose the application of an MCDA tool to rank ED pesticides based on their impact on reproductive health among the Costa Rican population. This ranking will help identify the most relevant ED pesticides to which the population may be exposed, thereby guiding future research, as well as informing management strategies, environmental policies, and regulatory decisions.

## Materials and methods

### Pesticide exposure analysis

**Fig. 1:**
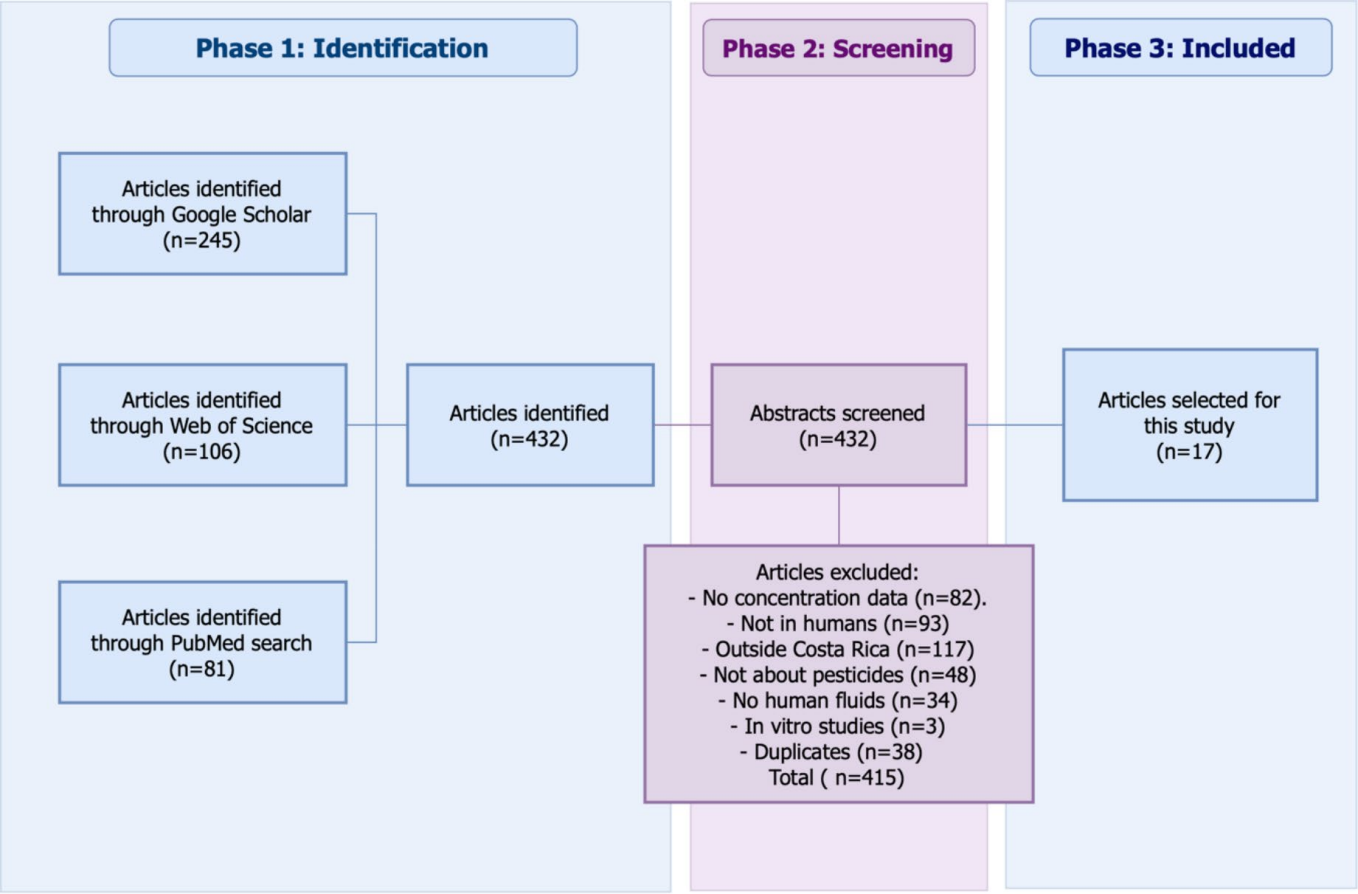
Article selection process to assess pesticide exposure in the Costa Rican population

To assess pesticide exposure in the Costa Rican population, reports on the presence of pesticides in human fluids from the past 25 years were analyzed. A comprehensive literature search was conducted in the PubMed and Web of Science databases using the keywords “pesticides” and “Costa Rica,” combined with the Boolean operator “AND.” The search was restricted to studies published between January 2000 and September 2024. On PubMed, the filter “humans” on species was applied, and on Web of Science, the search was refined by selecting the following filters: “Costa Rica” for Countries/Regions, and “herbicides, pesticides & ground poisoning”; “molecular & cell biology – DNA damage”; “crop protection”; “contamination & phytoremediation” and “urology & nephrology – general” for Citation Topic Meso. To include relevant literature in Spanish, the equivalent keywords “plaguicidas,” “Costa Rica,” and “disruptores endocrinos” were used, applying the same search strategy across both databases. Studies were included in the analysis if they reported pesticide concentrations in human fluids from Costa Rica. A total of 17 out of 432 peer-reviewed articles were selected for this study (Fig. 1). From these articles, a list of detected pesticides and their corresponding concentrations was compiled. For each pesticide, the internal concentration was calculated as the average of the concentrations of metabolites or parent compounds present in blood or urine with separate calculations performed for children and adults, as reported in the selected studies, along with their respective standard deviations.

Calculations were performed using R Studio 4.4.2 (R Core Team, 2024). Metabolites associated with more than one pesticide were excluded from the average calculation. Moreover, the highest reported concentration for each pesticide across all the selected papers was identified and recorded. The IUPAC name and the CAS number for each pesticide were obtained from the Pesticide Properties DataBase (PPDB) (Lewis et al., 2016). Subsequently, the identified pesticides were evaluated using MCDA to assess their potential impact on human reproductive health. This process required calculating the non-carcinogenic risk for each pesticide.

### Risk assessment

The risk assessment of the identified pesticides included an evaluation of the non-carcinogenic risks for adults, calculated using the hazard quotient (HQ) method as described in previous studies (Engelhardt et al., 2022; Yusà et al., 2022). Briefly, the HQ corresponds to the ratio of the internal concentration of pesticides in blood or urine, representing exposure, to the acceptable level reported as internal concentrations in the corresponding human fluid. The internal concentration for each pesticide is the average concentration (µg/L) reported in blood or urine across the selected studies. The acceptable level corresponds to established human biomonitoring guidance values (HBM-GVs), below which there is no risk of adverse effects. When HBM-GVs were unavailable, values from pesticides in the same group were used. If no values from the same pesticide group were available, the average of the available values for the identified pesticides was applied. The HBM-GVs were obtained from the German Human Biomonitoring Commission (2022). Blood HBM-GVs were converted to wet weight for comparison using a default value of 0.006656 g lipid/mL, calculated based on a lipid percentage of 0.65% and a human serum density of 1.024 g/mL, as reported in previous studies (Engelhardt et al., 2022).

Since most subjects in the included studies on the pesticide exposure analysis were plantation workers or individuals living near plantations, an additional hazard quotient specific to plantation workers (HQ_W_) was calculated, following methodologies from previous studies (Morales-Mora et al., 2022; Papadakis et al., 2015), with adaptations to align with the data obtained in this study. Here, HQ_W_ corresponds to the ratio of the internal concentration of pesticides in blood or urine, representing exposure, to the estimated absorbed permissible level of pesticide exposure for workers. The absorbed permissible level was estimated using Eq. 1.

1 $$\text{Absorbed permissible level}= \frac{\text{AOEL}\times \text{BW}\times \text{EF}\times \text{ED}\times \text{Dabs}}{\text{Volume}}$$

where:

AOEL = Acceptable operator exposure level (µg/kg BW/day).

BW = Estimated adult body weight (kg)—80.9 kg (Morales-Mora et al., 2022).

EF = Exposition frequency (days/year)—200 days/year (Morales-Mora et al., 2022).

ED = Exposition duration (years)—55 years (from 15 years old to 70 years old).

Dabs = Dermal absorption (%).

Volume = Estimated adult blood or urine volume (L)—5 L for blood (Parra, 2011) or 1.75 L for urine (Alessio et al., 1985; Knuiman et al., 1986; Lee et al., 2009).

Inhalation absorption was not considered in the calculation since its contribution to the internal concentration is minimal (Zemmouri et al., 2022). AOEL values and percentage of dermal absorption were obtained from the PPDB (Lewis et al., 2016) and the literature.

### Analytical hierarchy process

The analytical hierarchy process (AHP) was selected for the MCDA as it is part of a family of decision-making tools designed for ranking problems, such as the one presented in this study. Moreover, AHP requires a moderate level of modeling effort to perform the analysis while providing high-quality results. It involves assessing the relative importance of different decision alternatives based on selected criteria, thereby integrating evidence-based data into the decision-making process (Ishizaka & Nemery, 2013; Saaty, 1990). The AHP was applied as described by Lizano-Fallas and collaborators (Lizano-Fallas et al., 2023, 2024) with some modifications. Briefly, the problem was structured as a hierarchy with three levels: (1) the overall goal—ranking the pesticides identified in the exposure analysis according to their relevance to human reproductive health; (2) the criteria contributing to the overall goal; and (3) the alternatives, i.e., the pesticides identified in the exposure analysis. A pairwise comparison of the selected criteria was then conducted individually by all authors, based on their expertise. Each author assessed the relative importance of each criterion in comparison to the others, using a scale from 1 to 9. A score of 1 indicated equal contribution of the criteria to the goal, while a score of 9 indicated extreme importance of one criterion over another. For each author’s matrix, the consistency of the pairwise comparisons was evaluated, with an acceptable consistency threshold of up to 10% (Saaty, 1990). The group result for the priority vector, incorporating all authors’ judgments and the average AHP group consensus, was calculated.

Finally, the global priorities of the identified pesticides were established. To achieve this, the authors collectively assigned a semi-qualitative scale ranging from 1 to 9 for each criterion, where 1 represented the least important value and 9 the most important (Supplementary Table 1). Unlike the classic AHP scale, in which 1 indicates equivalence, here, 1 signified the lowest level of importance. This scale enabled the conversion of quantitative or qualitative values into comparable scores, which were then used to standardize comparisons. Using this scale, the authors conducted pairwise comparisons for each pesticide based on the available data for each criterion (Supplementary Table 6). To enhance accuracy, consistency, and efficiency, the process was implemented in an Excel spreadsheet, allowing pre-calculation of comparison values to be encoded in the AHP matrix following the defined AHP scale rules. This approach simplified pairwise comparisons and ensured a consistency ratio (CR) below 10%, thereby maintaining reliable and aligned results. Pairwise comparisons between pesticides were made by subtracting the score of the second pesticide from that of the first. The resulting values were adjusted to fit the AHP scale: positive or zero results were increased by 1, while negative results were decreased by 1. This adjustment method structured the relative priorities between pesticides. The final result determined which of the two pesticides was more important and in what proportion. The sign of the result indicated the most important pesticide: positive values gave greater importance to the first pesticide, while negative values favored the second. The magnitude of the result determined the proportion of importance. Pesticides were ranked based on their global priority, with those having lower rank numbers considered more relevant for their potential impact on human reproductive health.

All pairwise comparisons and calculations of priority vectors, CR, and group consensus were done using the online software AHP-OS (Goepel, 2018).

## Results and discussion

### Pesticide exposure analysis

From the pesticide exposure analysis, which included 17 human biomonitoring studies, a total of 13 pesticides were identified, five of which were also analyzed in children. Pesticide exposure was monitored through urine or blood samples, analyzing either the parent compounds or their metabolites using liquid or gas chromatography. The limited number of studies may be partly attributed to the sophisticated techniques required and insufficient funding (Yusa et al., 2015; Zúñiga-Venegas et al., 2022), a situation similarly observed in other countries in the region (Barrón Cuenca et al., 2024; Zúñiga-Venegas et al., 2022). Moreover, in Costa Rica, between 2010 and 2015, it was prohibited to conduct studies involving humans as subjects, which further limited research opportunities during that period (Householder et al., 2019). As a result of this prohibition, there are currently few human biomonitoring studies available in the country, most of which were conducted in limited geographic areas and several years ago. This historical restriction has contributed to gaps in the evidence, despite Costa Rica being considered a regional leader in this field. However, among Central American countries, Costa Rica has conducted more studies on pesticide exposure using biomonitoring compared to its neighbors. For instance, Nicaragua has reported four studies, while El Salvador has only one (Zúñiga-Venegas et al., 2022).

In the 17 studies included in this analysis, in adults, nine pesticides were detected in urine and five in blood, while in children, all five pesticides were monitored in urine. Pyrimethanil exhibited the highest mean concentration in urine for both adults (375.41 µg/L) and children (445.67 µg/L), followed by thiabendazole (301.43 µg/L in adults) and mancozeb (108.26 µg/L in adults). For children, 2,4-D (2,4-dichlorophenoxyacetic acid) ranked second, with a mean concentration of 146.85 µg/L. (Table 1). The most frequently reported pesticides were mancozeb (11 studies), chlorpyrifos (10), pyrimethanil (8), 2,4-D (7), and thiabendazole (6). The remaining eight pesticides were each reported in one study. Among the most frequently reported pesticides, chlorpyrifos (Barrón Cuenca et al., 2019, 2020; Filippi et al., 2021; Varona-Uribe et al., 2016), pyrimethanil (Barrón Cuenca et al., 2019, 2020), 2,4-D (Barrón Cuenca et al., 2020; Smpokou et al., 2019), and thiabendazole (Barrón Cuenca et al., 2019, 2020) have also been detected in human fluids in other Latin American countries, including Bolivia, Argentina, Colombia, and Nicaragua. While mancozeb, chlorpyrifos, 2,4-D, and glyphosate account into the most studied pesticides in human biomonitoring studies in Europe (RPA et al., 2017).

**Table 1:** Identified pesticides and reported concentrations in the pesticide exposure analysis

Pesticide	IUPAC name	CAS number	Pesticide type	Pesticide group	Sample	Concentration (µg/L), adults*	Concentration (µg/L), children*	Highest reported concentration (µg/L)
Mancozeb	Manganese ethylenebis(dithiocarbamate) (polymeric) complex with zinc salt	8018–01–7	Fungicide, insecticide	Carbamate	Urine	108.26 ± 75.13 (8)	50.54 ± 22.69 (2)	127.38
					Blood	52.03 ± 2.85 (4)	-	56.3
Pyrimethanil	*N*-(4,6-Dimethylpyrimidin- 2-yl)aniline	53112–28 - 0	Fungicide	Anilinopyrimidine	Urine	375.41 ± 298.59 (8)	445.67 (1)	946
Thiabendazole	2-(Thiazol- 4-yl)benzimidazole	148–79 - 8	Fungicide	Benzimidazole	Urine	301.43 ± 161.56 (6)	20.26 (1)	491
Chlorpyrifos	*O*,*O*-Diethyl *O*− 3,5,6-trichloro- 2-pyridyl phosphorothioate	2921–88 - 2	Insecticide	Organophosphate	Urine	53.02 ± 30.44 (8)	22.76 ± 10.94 (4)	101.65
2,4-D	(2,4-dichlorophenoxy)acetic acid	94–75 - 7	Herbicide	Phenoxy	Urine	58.18 ± 28.77 (7)	146.85 (1)	146.85
Glyphosate	*N*-(Phosphonomethyl)glycine	1071–83 - 6	Herbicide	Organophosphate	Urine	2.55 ± 2.67 (2)	-	4.43
Propineb	Polymeric zinc propylenebis	12,071–83 - 9	Fungicide	Carbamate	Urine	5.67 (1)	-	5.67
Paraquat	1,1′-Dimethyl- 4,4′-bipyridinium	4685–14 - 7	Herbicide	Quaternary ammonium	Urine	5.09 ± 3.59 (3)	-	8.96
DDT	1,1,1-Trichloro- 2,2-bis(4-chlorophenyl)ethane	50–29 - 3	Insecticide	Organochloride	Blood	> 0.05 (1)	-	> 0.05
Dieldrin	(1R,4S,4aS,5R,6R,7S,8S,8aR)− 1,2,3,4,10,10-Hexachloro- 1,4,4a,5,6,7,8,8a-octahydro- 6,7-epoxy- 1,4:5,8-dimethanonaphthalen	60–57 - 1	Insecticide	Cyclodiene	Blood	> 0.25 (1)	-	> 0.25
Chlorothalonil	Tetrachloroisophthalonitrile	1897–45 - 6	Fungicide	Chloronitrile	Blood	16.10 (1)	-	16.1
Lindane	1α,2α,3β,4α,5α,6β-Hexachlorocyclohexane	58–89 - 9	Insecticide	Organochloride	Blood	> 0.1 (1)	-	> 0.1
Tebuconazole	(RS)− 1-*p*-Chlorophenyl- 4,4-dimethyl- 3-(1*H*− 1,2,4-triazol- 1-ylmethyl)pentan- 3-ol	107,534–96 - 3	Fungicide	Triazole	Urine	45.17 (1)	-	45.17

*Concentration is presented as the mean of the reported concentrations across the studies included in the analysis, with the corresponding standard deviation. The number of studies reporting the pesticide is indicated in parentheses

The populations studied included elderly individuals (1 study), pregnant women (11), children (3), and farmworkers (2). Geographically, participants were from the entire country (2 studies) or specifically from the provinces of Alajuela (1) and Limón (14). Most studies focus on individuals living or working near banana plantations where pesticides are frequently applied. These areas are often classified as contaminated sites due to the high frequency of pesticide use. Detailed information from these studies is provided in Supplementary Information Table 3. In banana plantations, mancozeb is the main active ingredient in the current fungicide program. Other commonly used pesticides for this crop include pyrimethanil, thiabendazole, and chlorpyrifos (Brühl et al., 2023). Therefore, it is not surprising that these pesticides appear on the list as the most frequently reported.

Compared to a similar study conducted in Sweden (Engelhardt et al., 2022), the pesticide DDT (1,1,1-trichloro- 2,2-bis(4-chlorophenyl)ethane) showed similar concentrations in blood. In the analysis presented here, only one study reported this pesticide, with most participants (60%) being individuals who had not reported occupational exposure (Steenland et al., 2014). Although DDT was banned in Costa Rica in 1999 (SFE, 2024a) and in Sweden 30 years earlier (Hayes, 1969), its persistence in the environment allows it to bioaccumulate in humans, posing potential health concerns (Devi, 2020). Furthermore, of the seven pesticides identified in the Swedish study, only DDT was common to this Costa Rican study. This finding is unsurprising, given the differences in agricultural production between the two countries. Consequently, the pesticides to which populations are exposed differ, and the demand for insecticides is significantly lower in Sweden compared to many other countries (Hayes, 1969).

### Risk assessment

The HQ and the HQw were calculated to assess the risk associated with the identified pesticides. When the HQ (or HQw) is lower than 1, the risk of adverse effects is considered low. However, if the HQ (or HQw) is equal or higher than 1, the exposure may pose a risk of adverse effects. In this study, all HQ (or HQw) values were below 1 (Table 2), indicating a low risk. However, it is important to note that low HQ (or HQw) values do not guarantee complete safety, as they may still contribute to cumulative or long-term adverse health effects (Ali et al., 2021). Similar results have been observed in previous studies conducted in Spain (Yusà et al., 2022). These findings could be influenced by the fact that only two out of the 17 analyzed studies included follow-up sampling to monitor pesticide concentrations over time. In one of these cases, the follow-up was limited, with the first sample consisting of urine collected from the mother during pregnancy and the second consisting of urine from the children at 5 years old. This limitation is significant because relying on a single sample collected at a specific time point may not provide a comprehensive representation of the individual’s exposure over time. Results can vary depending on the timing of sample collection, especially in cases of chronic exposure to pesticides with short biological half-lives or high variability among individuals, which are commonly used in Latin America (Zúñiga-Venegas et al., 2022).

**Table 2:** Hazard quotients (HQ) and worker hazard quotients (HQw) of pesticides identified in different biological fluids

Pesticide	Sample	HQ	HQw
Mancozeb	Urine	0.0180	0.01106
Mancozeb	Blood	0.8985	0.01519
Pyrimethanil	Urine	0.0626	0.00003
Thiabendazole	Urine	0.0502	0.00085
Chlorpyrifos	Urine	0.0252	0.00083
2,4-D	Urine	0.0055	0.00028
Glyphosate	Urine	0.0005	0.00001
Propineb	Urine	0.0009	0.00885
Paraquat	Urine	0.0008	0.00500
DDT	Blood	0.0009	0.0000001
Dieldrin	Blood	0.0043	0.00012
Chlorothalonil	Blood	0.2780	0.00494
Lindane	Blood	0.0017	0.00001
Tebuconazole	Urine	0.0075	0.00002

It is important to note that while the detected values may be low, some of the identified pesticides, such as DDT, as has been mentioned before, have been banned for a long time and should no longer be present (SFE, 2024a). However, these pesticides possess properties such as high lipophilicity and a long half-life, which enable them to bioaccumulate and ultimately impact human health by affecting systems like the reproductive or cardiovascular systems, or by increasing the risk of cancer (Devi, 2020). Thus, the low detected values could be attributed to the persistence of these compounds, either from past exposures or ongoing environmental contamination.

The low HQ and HQw values might also be shaped by limitations in the analyzed studies, particularly their small sample sizes (Steenland et al., 2014). For instance, 16 of the 17 reviewed studies included a maximum of 400 subjects, while only one study involved a cohort of 2000 individuals from Sweden, which was compared with a sample from Costa Rica (Krais et al., 2023). Consequently, the limited sample sizes in these studies could impact the accuracy of the calculated HQ values. This aligns with previous research that has questioned the validity of pesticide exposure studies in humans due to insufficient sample sizes, which result in a lack of statistical power (London et al., 2010).

Additionally, assessing the risk of pesticides is challenging due to the difficulty in obtaining enough information, as not all pesticides have been evaluated for exposure toxicity (Engelhardt et al., 2022). The calculations of HQ and HQw had to rely on acceptable levels derived from internal blood concentrations as described in previous studies (Engelhardt et al., 2022), limiting the number of available values for all identified pesticides. Therefore, it was necessary to use the values reported for the chemical group to which the pesticide belonged or to calculate the average of the values reported for other pesticides (Supplementary Tables 4 and 5).

Also, some of the studies reviewed only report the limit of detection (LOD), which does not represent the actual concentration of the pesticide in human fluids. This limitation may result in an underestimation of exposure levels and their potential health risks. Notably, the HQ values for mancozeb and chlorothalonil in blood are close to the threshold, which is concerning not only because they are classified as endocrine disruptors but also due to reports indicating their potential to negatively affect human reproduction (Lewis et al., 2016).

The HQw values are lower than the HQ values for all pesticides. This difference may be attributed to HQw exclusively assessing occupational exposure, which represents only a subset of total exposure pathways. Remarkably, mancozeb once again exhibits the highest value among the pesticides. This can be explained by its extensive use in banana cultivation, a common agricultural practice in Costa Rica (Brühl et al., 2023). Mancozeb is particularly relevant due to its potential for long-term exposure effects in agricultural workers, who are frequently exposed to this pesticide in their daily work. While the HQw value for mancozeb is below 1, its high use and the continuous exposure of workers make it a critical point of concern due to its reproductive toxicity and potential for endocrine disruption (Runkle et al., 2017). Similarly, chlorothalonil, another pesticide with a relatively high HQ, is commonly used in banana plantations and has been linked to liver and kidney toxicity, as well as being classified as a probable human carcinogen by the International Agency for Research on Cancer (IARC). Although its HQw value is also below 1, the pesticide’s persistence in the environment and its potential for bioaccumulation mean that chronic exposure could still pose a health risk, especially for workers and residents living near plantations (Sun et al., 2022).

Although the HQ and HQw values obtained are relatively low, the population in Costa Rica living near plantations remains at risk of health problems due to the extensive use of endocrine-disrupting pesticides in these crops (Brühl et al., 2023). The high frequency of pesticide application further exacerbates this risk, increasing health hazards for people living or working near these agricultural areas (Brühl et al., 2023).

### Analytical hierarchy process

Based on the authors’ expertise, eight criteria were selected to prioritize the identified pesticides. Extensive research was conducted to ensure the validity and consistency of the selected criteria, as well as to confirm the availability of data for each pesticide (Table 3 and Supplementary Table 6). The criteria *non-carcinogenic risk*, *number of adverse effects reported on reproduction*, and *classification as an endocrine disruptor* together account for nearly three-quarters of the total importance across all criteria. This prioritization is logical, as these factors are directly related to human health and reproduction. The *non-carcinogenic risk* criterion reflects the actual concentration of a substance in bodily fluids (e.g., blood and urine), which indicates human exposure and accounts for the substance’s toxicity, calculated using toxicological data. Meanwhile, *endocrine disruptor status* refers to substances associated with hormonal disorders, which can exacerbate adverse reproductive effects. Next is the *No Observed Effect Concentration* (NOEC) criterion, which holds a score of 9%, indicating intermediate but relatively low importance. This ranking is consistent, as the criterion relates to chronic exposure in the bioindicator *Daphnia magna*, a species commonly used in ecotoxicology to evaluate the effects of harmful chemical substances on reproduction. While the NOEC is not directly linked to human health, impacts on the reproduction of aquatic invertebrates, such as *D. magna*, could indicate potential risks to human reproductive health.

**Table 3:** Description of the criteria used to prioritize the identified pesticides via the AHP method

Criteria	Source	Description	Selection support	Priority vector
Non-carcinogenic risk	Calculated according to the data obtained from the systematic review	The higher the non-carcinogenic risk, estimated by the hazard quotient method, the more relevant the pesticide becomes	(Tsaboula et al., 2016; Wesseling et al., 1999)	0.25
Number of adverse effects reported on reproduction	PPDB (Lewis et al., 2016)	Pesticide is more relevant if it has a higher number of reported negative effects on human reproduction in the PPDB	(Mostafalou & Abdollahi, 2017; Perry, 2008; Sifakis et al., 2011)	0.24
Classification as an endocrine disruptor	PPDB (Lewis et al., 2016)	Pesticide is more relevant if it is classified as an endocrine disruptor	(McKinlay et al., 2008; Mnif et al., 2011)	0.22
NOEC—chronic test in *D. magna* (reproduction test)	PPDB (Lewis et al., 2016)	The NOEC value and the classification/interpretation (low, moderate, high toxicity) will be used. The higher the toxicity, the more relevant the pesticide	(Tsaboula et al., 2016)	0.09
Number of studies in which it was reported	Systematic review	Pesticide becomes more relevant as the number of studies reporting it in the review increases	(Carazo-Rojas et al. 2018; Ramírez-Morales et al., 2021; Echeverría-Sáenz et al., 2021)	0.06
International regulatory status	Pesticide Action Network (PAN) (PAN, 2022)	Pesticide is more relevant if it appears on The Consolidated List of Banned Pesticides by the PAN	(Donley, 2019; Roșca et al., 2023)	0.05
Costa Rican regulatory status	Costa Rica State Phytosanitary Service (SFE) (SFE, 2024b, 2024a)	Pesticide is more relevant if it appears on the banned or restricted lists of the Costa Rican SFE	(Brühl et al., 2023)	0.05
Estimated use	Ministry of Agriculture and Livestock, Costa Rica (SFE, 2022)	The higher the estimated use of the pesticide, the more relevant it becomes. The estimated total pesticide consumption is determined by subtracting pesticide exports from imports	(Bravo-Durán et al., 2015; de la Cruz et al., 2014)	0.04
Consistency ratio	0.02			
Consensus	0.75			

Finally, the following criteria were considered: *number of studies in which the pesticide was reported* (6%), *international regulatory status* (5%), *regulatory status in Costa Rica* (5%), and *estimated use* (4%). Although these criteria have the lowest importance, they serve as secondary factors that support the interpretation of risks. The *international regulatory status* and *Costa Rican regulatory status* provide insight into how a substance is perceived as problematic on both global and local scales. International regulations were included to offer a broader perspective, particularly when local policies may not fully address the risks. Despite regulatory efforts in some countries, many pesticides with recognized harmful effects continue to be widely used in agriculture across much of the world, underscoring the challenges of balancing agricultural needs with health and environmental protection.

Once the priority vector of the criteria was determined, the alternatives, i.e., the 13 pesticides, were ranked based on the data for each criterion and the collective judgment of the authors. Mancozeb ranked highest in its potential impact on human reproductive health (14.8%), as it has the highest HQ and the lowest NOEC. Mancozeb is also an endocrine disruptor, has one reproductive effect reported in the PPDB, and is the pesticide with the highest number of studies reported. Dieldrin followed in second place (12.1%). Although it does not have a very high HQ and is classified as low risk on the scale proposed by the authors, it is an endocrine disruptor and the only pesticide with two reproductive effects documented in the PPDB—the highest number recorded. Chlorothalonil ranked third (12.0%) due to its second-highest HQ, its classification as an endocrine disruptor, and one reported reproductive effect. It also has the third-lowest NOEC among the pesticides evaluated. Pesticides such as lindane, DDT, and 2,4-D, which demonstrated low to moderate risk, are endocrine disruptors with one reported reproductive effect each. These pesticides occupied middle positions on the list, with approximately 7% each.

The top six pesticides identified are classified as EDs with similar modes of action, primarily affecting the thyroid hormone function by altering synthesis, activity, or concentrations (Baratzhanova et al., 2024; Corrales Vargas et al., 2022; Sang et al., 1999), except for chlorothalonil, which interferes with estrogen production (Zhang et al., 2019). The remaining pesticides, which ranked lowest in the prioritization, are not locally restricted or banned, except for paraquat, which is banned (Table 4 and Supplementary Tables 2 and 7).

**Table 4:** Overall ranking of pesticides based on global priority weights

Pesticide	Global priority	Consistency ratio
Mancozeb	0.1479	0.03
Dieldrin	0.1209	
Chlorothalonil	0.1200	
Chlorpyrifos	0.0941	
Lindane	0.0750	
DDT	0.0732	
2,4-D	0.0721	
Pyrimethanil	0.0649	
Thiabendazole	0.0593	
Tebuconazole	0.0588	
Glyphosate	0.0417	
Paraquat	0.0367	
Propineb	0.0356	

These results underscore the importance of considering a comprehensive set of criteria when prioritizing pesticides for further study regarding their impact on human reproduction. A global perspective on each pesticide is essential to more accurately evaluate their potential reproductive health effects. For instance, relying solely on the non-carcinogenic risk criterion would only reflect general toxicity based on pesticide concentrations in human matrices (e.g., blood and urine). Incorporating additional criteria broadens the scope to include other aspects that may indirectly affect reproduction. Together, these criteria provide a holistic view, offering insights into how criteria collectively contribute to prioritizing pesticides regarding their reproductive health risks.

## Conclusions

Human biomonitoring studies on pesticides are limited in Costa Rica, although the country leads the Central American region in the number of such studies. Information gaps still need to be addressed, such as relevant pesticides not included in past research or reports on internal pesticide residues in the general population, rather than solely in workers or residents of agricultural areas. Establishing biomonitoring programs and public health surveillance systems is recommended to generate updated data and support the development of evidence-based prevention policies and interventions, as the findings of this study are more than 10 years old.

Additionally, future studies should consider that farmers often mix pesticides when spraying their crops, frequently without using the necessary personal protective equipment. Therefore, the association with adverse effects should be analyzed from an exposomic perspective rather than focusing on individual pesticides.

## Supplementary Information

Below is the link to the electronic supplementary material.

ESM 1 (PDF 767 KB)

## Data Availability

All data on the pesticide exposure analysis collected from the literature search and all data for each identified pesticide for each criterion and the judgments for the analytical hierarchy process that support the findings of this study are included within this paper and its Supplementary Information files.
