# Supplementary material for: Endocrine-disrupting pesticide exposure relevant to reproductive health: a case study from Costa Rica
Source: Environ Monit Assess. 2025 Apr 16;197(5):559. doi: 10.1007/s10661-025-14011-8 (PMC12003610; doi:10.1007/s10661-025-14011-8)
Supplement: Supplementary file 1 — (PDF 767 KB) [file 10661_2025_14011_MOESM1_ESM.pdf]

Supplementary Table 1. Semi-qualitative scale ranging from 1 to 9 for each criterion.

Criteria	Interpretation	AHP Score
Non-carcinogenic risk	Very High Risk (HQ ≥ 0.5)	9
	High Risk (0.1 ≤ HQ < 0.5)	8
	Moderate to High Risk (0.05 ≤ HQ < 0.1)	7
	Moderate Risk (0.01 ≤ HQ < 0.05)	6
	Low to Moderate Risk (0.005 ≤ HQ < 0.01)	5
	0.001 ≤ HQ < 0.005	4
	Very Low Risk (HQ < 0.001)	3
Number of negative effects reported on reproduction	Reported twice	9
	Reported once	6
	Possibly, status not identified	5
Classification as an endocrine disruptor	Yes, is an endocrine disruptor	9
	Possibly, status not identified	6
	No, is not an endocrine disruptor	1
	Not data found	5
NOEC - chronic test in D. magna (reproduction test)	High toxicity (NOEC < 0.01)	9
	Moderate toxicity (0.01 ≤ NOEC < 1)	6
	Low toxicity (NOEC ≥ 1)	3
	Unknown	5
Number of studies in which it was reported	Reported 11 times	9
	Reported 10 times	8
	Reported 8 times	7
	Reported 7 times	6
	Reported 6 times	5
	Reported once	1
International regulatory status (Countries where banned)	Quasi universally banned (≥100)	9
	Majorly Banned (50 to 99)	6
	Moderately Banned (10 to 49)	5

Criteria	Interpretation	AHP Score
	Slightly Banned (5 to 9)	3
	Rarely Banned (1 to 4)	2
Costa Rican regulatory status	Not Banned (0)	1
	Not banned or restricted	1
	Restricted	5
	Banned	9
Estimated use	Very high usage (> 1000000)	9
	High usage (100000 < Usage ≤ 1,000,000)	8
	Medium usage (10000 < Usage ≤ 100000)	7
	Not reported	5
	Low usage (1000 < Usage ≤ 10000)	6
	Banned	4

Supplementary Table 2. Database containing the information of each pesticide for each criterion

Pesticide	Number of studies in which it was reported	Fluids	Non-carcinogenic risk	Classification as an endocrine disruptor	Number of negative effects reported on reproduction	Estimated use (Kg)	International Regulatory Status	Costa Rican Regulatory Status	NOEC (mg l^{-1})
Mancozeb	11	Urine	0.018	Yes	1	3317216,88	Banned in 31 countries	Not banned or restricted	0.0073
		Blood	0.8985						
Pyrimethanil	8	Urine	0.0626	Possibly, status not identified	0	-8008,18	Not banned	Not banned or restricted	0.94
Thiabendazole	6	Urine	0.0502	Not data found	0	4485,20	Banned in 1 country (Norway)	Not banned or restricted	0.042
Chlorpyrifos	10	Urine	0.0252	Yes	0	147258,19	Banned in 39 countries	Restricted	0.0046
2,4-D	7	Urine	0.0055	Yes	1	840052,66	Banned in 5 countries	Not banned or restricted	46.2
Glyphosate	1	Urine	0.0005	Possibly, status not identified	1	369710,90	Banned in 4 countries	Not banned or restricted	12.5
Propineb	1	Urine	0.0009	Possibly, status not identified	0	70196,00	Banned in 31 countries	Not banned or restricted	0.015
Paraquat	1	Urine	0.0008	No	0	157245,28	Banned in 58 countries	Restricted	0.12
DDT	1	Blood	0.0009	Yes	1	Banned	Banned in 157 countries	Banned	Unknown
Dieldrin	1	Blood	0.0043	Yes	2	Banned	Banned in 152 countries	Banned	Unknown

Pesticide	Number of studies in which it was reported	Fluids	Non-carcinogenic risk	Classification as an endocrine disruptor	Number of negative effects reported on reproduction	Estimated use (Kg)	International Regulatory Status	Costa Rican Regulatory Status	NOEC (mg l^{-1})
Chlorothalonil	1	Blood	0.278	Yes	1	73036,44	Banned in 34 countries	Banned	0.009
Lindane	1	Blood	0.0017	Yes	1	Not reported	Banned in 141 countries	Banned	54
Tebuconazole	1	Urine	0.0075	Yes	Unknown	11 565,46	Banned in 1 country (Palestine)	Not banned or restricted	0.01

Supplementary Table 3. Information retrieved from the studies included in the pesticide exposure analysis

ID	Reference	Study group	Subjects	Sample	Cohort	Year	Place	Province	Pesticide/ Agent	Concentration
1	Steenland K, Mora AM, Barr DB, Juncos J, Roman N, Wesseling C. Organochlorine chemicals and neurodegeneration among elderly subjects in Costa Rica. Environ Res. 2014;134:205-209. doi:10.1016/j.envres.2014.07.024	Elderly Costa Ricans	89 subjects, 36 (40%) had reported occupational pesticide exposure and 53 (60%) had not	Blood	Not indicated	2012	Costa Rica	Whole Costa Rica	β-HCH (lindane)<br><br>Dieldrin<br><br>p,p'–DDT (DDT)<br><br>p,p'–DDE (a metabolite of DDT)	100% above LOD (0,1 µg/L)<br><br>57% above LOD (0,25 µg/L)<br>75% above LOD (0,05 µg/L)<br><br>93% above LOD (0,05 µg/L)
2	Corrales Vargas A, Peñaloza Castañeda J, Rietz Liljedahl E, et al. Exposure to common-use pesticides, manganese, lead, and thyroid function among pregnant women	Women aged >15 years with a gestational age of <33 weeks	400	Urine	Environmental Health (ISA) study	Between march 2010 and june 2011	In a banana growing area in the Northern Caribbean of Costa Rica in Matina county	Limón	Ethylenethiourea (ETU; a metabolite of mancozeb)<br>3-hydroxy-pyrimethanil (OHP; a metabolite of	251 µg/L<br><br>946 µg/L

ID	Reference	Study group	Subjects	Sample	Cohort	Year	Place	Province	Pesticide/ Agent	Concentration
	from the Infants' Environmental Health (ISA) study, Costa Rica. Sci Total Environ. 2022;810:151288. doi:10.1016/j.scitotenv.2021.151288								pyrimethanil)	
									5-hydroxy-thiabendazole (OHT; a metabolite of thiabendazole)	491 µg/L
									3,5,6-trichloro-2-pyridinol (TCPy; a metabolite of the OP chlorpyrifos)	50.0 µg/L
									2,4-dichlorophenoxyacetic acid (2,4-D; parent compound)	39.7 µg/L
									3-(2,2-dichlorovinyl)-2,2-Dimethylcyclopropane carboxylic acid	45.8 µg/L

ID	Reference	Study group	Subjects	Sample	Cohort	Year	Place	Province	Pesticide/ Agent	Concentration
									(DCCA; a metabolite of pyrethroid insecticides)	
									3-phenoxybenzoic acid (3-PBA; a metabolite of pyrethroid insecticides)	32.6 µg/L
				Hair					Manganese (mancozeb)	53.2 µg/L
				Blood					Manganese (mancozeb)	50.6 µg/L
									Lead level	6,66 µg/L
3	Mora AM, Baker JM, Hyland C, et al. Pesticide exposure and cortical brain activation among farmworkers in Costa Rica. NeuroToxicology	Farmworkers aged 18 or older	48	Urine	Not indicated	2016	Zarcero County	Alajuela	3,5,6-trichloro-2-pyridinol (TCPy; a metabolite of the OP chlorpyrifos)	101.65 µg/L
									3-phenoxybe	16.19 µg/L

ID	Reference	Study group	Subjects	Sample	Cohort	Year	Place	Province	Pesticide/ Agent	Concentration
	. 2022;93:200-210. doi:10.1016/j.neuro.2022.10.004								nzoic acid (3-PBA; a metabolite of pyrethroid insecticides)	
									4-fluoro-3-phenoxybenzoic acid (4F3PBA; a metabolite of pyrethroid insecticides)	0.15 µg/L
										34.86 µg/L
									Chloro-3,3,3-trifluoro-1-propene-1-yl (CFCA; a metabolite of pyrethroid insecticides)	1.27 µg/L
									Ethylenethiourea (ETU; a	44.91 µg/L

ID	Reference	Study group	Subjects	Sample	Cohort	Year	Place	Province	Pesticide/ Agent	Concentration
									metabolite of mancozeb)	
									propylenethiourea (PTU; a metabolite of propineb)	5.67 µg/L
									5-hydroxy-thiabendazole (OHT; a metabolite of thiabendazole)	0.63 µg/L
									3-hydroxy-pyrimethanil (OH-P; a metabolite of pyrimethanil)	559.32 µg/L
									t-butyl-hydroxy tebuconazole (TEB-OH; a metabolite of	45.17 µg/L

ID	Reference	Study group	Subjects	Sample	Cohort	Year	Place	Province	Pesticide/ Agent	Concentration
									tebuconazole)	
									2,4-dichlorophenoxyacetic acid (2,4-D; parent compound)	9.10 µg/L
									Glyphosate (GLY; parent compound)	4.43 µg/L
									aminomethylphosphonic acid (AMPA; a degration production of glyphosate)	0.66 µg/L
4	Mora AM, van Wendel de Joode B, Mergler D, et al. Maternal blood and hair manganese concentrations, fetal growth, and length of gestation in the	Women aged >15 years with a gestational age of <33 weeks	380	Blood	Environmental Health (ISA) study	Between March 2010 and June 2011	Living near banana plantations in Matina County	Limón	Manganese (mancozeb)	50.6 µg/L
				Hair					Manganese (mancozeb)	53.3 µg/g

ID	Reference	Study group	Subjects	Sample	Cohort	Year	Place	Province	Pesticide/ Agent	Concentration
	ISA cohort in Costa Rica. Environ Res. 2015;136:47-56. doi:10.1016/j.envres.2014.10.011									
5	Van Wendel de Joode B, Mora AM, Lindh CH, et al. Pesticide exposure and neurodevelopment in children aged 6–9 years from Talamanca, Costa Rica. Cortex. 2016;85:137-150. doi:10.1016/j.cortex.2016.09.003	Children aged 6-9	140	Urine	Not indicated	February and August 2007	Living near banana plantations and plantain farms in the Talamanca County	Limón	3,5,6-trichloro-2-pyridinol (TCPy; a metabolite of the OP chlorpyrifos)	26.8 μg/L
									3-phenoxybenzoic acid (3-PBA; a metabolite of pyrethroid insecticides)	20.4 μg/L
									Ethylenethiourea (ETU; a metabolite of mancozeb)	34.5 μg/L

ID	Reference	Study group	Subjects	Sample	Cohort	Year	Place	Province	Pesticide/ Agent	Concentration
6	Krais AM, Joode B van W de, Liljedahl ER, et al. Detection of the fungicide transformation product 4-hydroxychlorothalonil in serum of pregnant women from Sweden and Costa Rica. J Expo Sci Environ Epidemiol. 2023;34(2):270. doi:10.1038/s41370-023-00580-8	Pregnant Swedish women	1808	Blood	Autism and Prenatal Endocrine Disruptors (APED) study	1997–2015	Scania county, Sweden	Sweden	4-hydroxychlorothalonil (HCT; a metabolite of chlorothalonil)	4.1 µg/L
		Pregnant Costa Rican women	393	Blood	Environmental Health (ISA) study	Between March 2010 and June 2011	Matina County	Limón	4-hydroxychlorothalonil (HCT; a metabolite of chlorothalonil)	16.1 ug/L
7	Alhanti B, Joode B van W de, Martinez MS, et al. Environmental exposures	Women aged >15 years with a gestatio	259	Urine	Environmental Health (ISA) study	Between March 2010 and June 2011	Living near banana plantations in Matina County	Limón	Ethylenethiourea (ETU; a metabolite of mancozeb)	57.71 µg/L

ID	Reference	Study group	Subjects	Sample	Cohort	Year	Place	Province	Pesticide/ Agent	Concentration
	contribute to respiratory and allergic symptoms among women living in the banana growing regions of Costa Rica. Occup Environ Med. 2021;79(7):469. doi:10.1136/oemed-2021-107611	nal age of <33 weeks							3-hydroxy-pyrimethanil (OHP; a metabolite of pyrimethanil)	20.97 µg/L
									5-hydroxy-thiabendazole (OHT; a metabolite of thiabendazole)	299.96 µg/L
									3,5,6-trichloro-2-pyridinol (TCPy; a metabolite of the OP chlorpyrifos)	16.41 µg/L
									3-(2,2-dichlorovinyl)-2,2-Dimethylcyclopropane carboxylic acid (DCCA; a	30.32 µg/L

ID	Reference	Study group	Subjects	Sample	Cohort	Year	Place	Province	Pesticide/ Agent	Concentration
									metabolite of pyrethroid insecticides)	
									3-phenoxybenzoic acid (3-PBA; a metabolite of pyrethroid insecticides)	41.37 μg/L
									2,4-dichlorophenoxyacetic acid (2,4-D; parent compound)	39.41 μg/L
									1-HP (Polycyclic aromatic hydrocarbons)	18.22 μg/L
									2-OH-PH (Polycyclic aromatic hydrocarbons)	39.96 μg/L

ID	Reference	Study group	Subjects	Sample	Cohort	Year	Place	Province	Pesticide/ Agent	Concentration
8	Joode B van W de, Peñaloza-Castañeda J, Mora AM, et al. Pesticide exposure, birth size, and gestational age in the ISA birth cohort, Costa Rica. Environ Epidemiol. 2024;8(2):e290. doi:10.1097/EE9.0000000000000290	Women aged >15 years with a gestational age of <33 weeks	386	Urine	Environmental Health (ISA) study	Between March 2010 and June 2011	Living at <5 kilometers from a banana plantation in Matina County	Limón	Ethylenethiourea (ETU; a metabolite of mancozeb)	127.38 µg/L
									3-hydroxy-pyrimethanil (OHP; a metabolite of pyrimethanil)	368.55 µg/L
									3,5,6-trichloro-2-pyridinol (TCPy; a metabolite of the OP chlorpyrifos)	62.96 µg/L
										23.56 µg/L
									3-phenoxybenzoic acid (3-PBA; a metabolite of pyrethroid insecticide	16.96 µg/L

ID	Reference	Study group	Subjects	Sample	Cohort	Year	Place	Province	Pesticide/ Agent	Concentration
									s)	
									2,4-dichlorophenoxyacetic acid (2,4-D; parent compound)	79.76 µg/L
9	Giffin A, Hoppin JA, Córdoba L, et al. Pyrimethanil and chlorpyrifos air concentrations and pregnant women's urinary metabolites in the Infants' Environmental Health Study (ISA), Costa Rica. Environ Int. 2022;166:107328. doi:10.1016/j.envint.2022.107328	Women during pregnancy	448	Urine	Environmental Health (ISA) study	Between March 2010 and June 2011	In Matina County	Limón	3-hydroxy-pyrimethanil (OHP; a metabolite of pyrimethanil)	2.75 µg/L
									3,5,6-trichloro-2-pyridinol (TCPy; a metabolite of the OP chlorpyrifos)	4.27 µg/L.
10	Lee K, Park EK, Stoecklin-Marois M, et al. Occupational	Plantation workers	119 paraquat handlers and 54 non-handlers	Urine	Not indicated	May to December 2001	Five banana farms, ten coffee farms and one palm	Whole Costa Rica	Paraquat	Coffee: 5.74 µg/24 h (4.45 µg/L) Banana: 11.39

ID	Reference	Study group	Subjects	Sample	Cohort	Year	Place	Province	Pesticide/ Agent	Concentration
	paraquat exposure of agricultural workers in large Costa Rican farms. Int Arch Occup Environ Health. 2009;82(4):455-462. doi:10.1007/s00420-008- 0356-7						oil plantation			μg/24 h (8.96 μg/L) Palm oil: 2.19 μg/24 h (1.87 μg/L)
11	Mora AM, Córdoba L, Cano JC, et al. Prenatal Mancozeb Exposure, Excess Manganese, and Neurodevelopment at 1 Year of Age in the Infants' Environmental Health (ISA) Study. Environ Health Perspect. 2018;126(5):057007.	Women aged >15 years with a gestational age of <33 weeks	355	Urine	Environmental Health (ISA) study	Between March 2010 and June 2011	≤ 5 km from a banana plantation in Matina County	Limón	Ethylenethiourea (ETU; a metabolite of mancozeb)	127.4 μg/L
				Hair					Manganese (mancozeb)	53.3 μg/g
				Blood					Manganese (mancozeb)	50.6 μg/L

ID	Reference	Study group	Subjects	Sample	Cohort	Year	Place	Province	Pesticide/ Agent	Concentration
	doi:10.1289/EHP1955									
12	Mora, A. M., van Wendel de Joode, B., Mergler, D., Córdoba, L., Cano, C., Quesada, R., Smith, D. R., Menezes-Filho, J. A., Lundh, T., Lindh, C. H., Bradman, A., & Eskenazi, B. (2014). Blood and hair manganese concentrations in pregnant women from the infants' environmental health study (ISA) in Costa Rica. Environmental science & technology, 48(6), 3467–	Pregnant Costa Rican women	449	Blood	Environmental Health (ISA) study	Between March 2010 and June 2011	Matina County	Limón	Manganese (mancozeb)	56.3 μg/L
				Hair					Manganese (mancozeb)	53.3 μg/g

ID	Reference	Study group	Subjects	Sample	Cohort	Year	Place	Province	Pesticide/ Agent	Concentration
	3476. https://doi.org/10.1021/es404279r									
13	Joode B van W de, Mora AM, Córdoba L, et al. Aerial Application of Mancozeb and Urinary Ethylene Thiourea (ETU) Concentrations among Pregnant Women in Costa Rica: The Infants' Environmental Health Study (ISA). Environ Health Perspect. 2014;122(12):1321. doi:10.1289/ehp.1307679	Pregnant Costa Rican women	451	Urine	Environmental Health (ISA) study	Between March 2010 and June 2011	In Matina County	Limón	Ethylenethiourea (ETU; a metabolite of mancozeb)	2.9 µg/L
14	Conejo-Bolaños LD, Mora AM, Hernández-Bonilla D, et al. Prenatal current-	Women during pregnancy	355	Urine	Environmental Health (ISA) study	Between March 2010 and June 2011	Lived within 5 km of a banana plantation in the Costa Rica	Limón	3-hydroxy-pyrimethanil (OHP; a metabolite of	368.55 µg/L

ID	Reference	Study group	Subjects	Sample	Cohort	Year	Place	Province	Pesticide/ Agent	Concentration
	use pesticide exposure and children's neurodevelopment at one year of age in the Infants' Environmental Health (ISA) birth cohort, Costa Rica. Environ Res. 2024;249:118222. doi:10.1016/j.envres.2024.118222						Caribbean, in a rural area		pyrimethanil)	
									3,5,6-trichloro-2-pyridinol (TCPy; a metabolite of the OP chlorpyrifos)	62.96 µg/L
									3-(2,2-dichlorovinyl)-2,2-Dimethylcyclopropane carboxylic acid (DCCA; a metabolite of pyrethroid insecticides)	23.56 µg/L
									3-phenoxybenzoic acid (3-PBA; a metabolite of pyrethroid	16.96 µg/L

ID	Reference	Study group	Subjects	Sample	Cohort	Year	Place	Province	Pesticide/ Agent	Concentration
									insecticides)	
									2,4-dichlorophenoxyacetic acid (2,4-D; parent compound)	79.76 µg/L
									5-hydroxy-thiabendazole (OHT; a metabolite of thiabendazole)	339.00 µg/L
15	Mora AM, Hoppin JA, Córdoba L, et al. Prenatal pesticide exposure and respiratory health outcomes in the first year of life: Results from the infants' Environmental Health (ISA) study. Int J Hyg Environ Health.	Women aged >15 years with a gestational age of <33 weeks	93 women provided three samples during pregnancy, 222 women provided two samples, and 40 provided only one.	Urine	Environmental Health (ISA) study	Between March 2010 and June 2011	Living near banana plantations in Matina County	Limón	Ethylenethiourea (ETU; a metabolite of mancozeb)	127.38 µg/L
									3,5,6-trichloro-2-pyridinol (TCPy; a metabolite of the OP chlorpyrifos)	62.96 µg/L
									3-	16.96 µg/L

ID	Reference	Study group	Subjects	Sample	Cohort	Year	Place	Province	Pesticide/ Agent	Concentration
	2020;225:113474. doi:10.1016/j.ijheh.2020.113474								phenoxybenzoic acid (3-PBA; a metabolite of pyrethroid insecticides)	
									2,4-dichlorophenoxyacetic acid (2,4-D; parent compound)	79.76 µg/L
									3-(2,2-dichlorovinyl)-2,2-Dimethylcyclopropane carboxylic acid (DCCA; a metabolite of pyrethroid insecticides)	23.56 µg/L
									hydroxypyrimethanil (OH-PYR; a	368.55 µg/L

ID	Reference	Study group	Subjects	Sample	Cohort	Year	Place	Province	Pesticide/ Agent	Concentration
									metabolite of pyrimethanil)	
									5-hydroxythiabendazole (5-OH-TBZ; a metabolite of thiabendazole)	339.00 µg/L
16	Van Wendel de Joode B, Barraza D, Ruepert C, et al. Indigenous children living nearby plantations with chlorpyrifos-treated bags have elevated 3,5,6-trichloro-2-pyridinol (TCPy) urinary concentrations. Environ Res. 2012;117:17-26. doi:10.1016/j.en	Children aged 6–9 years	140	Urine	Not indicated	Between February and August 2007	In three villages Daytonia, Shiroles, Amubrë situated in the Caribbean lowlands of Talamanca	Limón	3,5,6-trichloro-2-pyridinol (TCPy; a metabolite of the OP chlorpyrifos)	Banana: 6.6 µg/L<br>Plantain: 26.8 µg/L
				Hand wash					Chlorphyrifos	Banana: 67 ng/both hands<br>Plantain: 52 ng/both hands
				Foot wash						Banana:108 ng/both feet<br>Plantain: 89 ng/both feet

ID	Reference	Study group	Subjects	Sample	Cohort	Year	Place	Province	Pesticide/ Agent	Concentration
	vres.2012.04.006			Children's mattress dust						Banana: 0.22 (0.01) µg/g Plantain: 34.9 µg/g
				Indoor dust						Banana: 1.2µg/g Plantain:0.10 µg/g
				Outside air (high volume)						Banana:4.9 ng/m^3 Plantain: -
				Outside air (passive sampler)						Banana: 9.3 ng/m^3 Plantain: -
17	Islam JY, Hoppin J, Mora AM, et al. Respiratory and allergic outcomes among 5-year-old children exposed to pesticides. Thorax. 2022;78(1):41. doi:10.1136/thoraxjnl-2021-218068	Children Among 5- year-old	303	Urine prenatal	Environmental Health (ISA) study	Between March 2010 and June 2011	Living within 5 km of banana plantations in Matina County	Limón	3,5,6-trichloro-2-pyridinol (TCPy; a metabolite of the OP chlorpyrifos)	62.96 µg/L
									Ethylenethiourea (ETU; a metabolite of mancozeb)	127.38 µg/L
									3-hydroxy-pyrimethan	368.55 µg/L

ID	Reference	Study group	Subjects	Sample	Cohort	Year	Place	Province	Pesticide/ Agent	Concentration
									il (OHP; a metabolite of pyrimethanil)	
									5-hydroxy-thiabendazole (OHT; a metabolite of thiabendazole)	339.00 µg/L
									2,4-dichlorophenoxyacetic acid (2,4-D; parent compound)	79.76 µg/L
									3-phenoxybenzoic acid (3-PBA; a metabolite of pyrethroid insecticides)	16.96 µg/L
									3-(2,2-dichlorovinyl)-2,2-	23.56 µg/L

ID	Reference	Study group	Subjects	Sample	Cohort	Year	Place	Province	Pesticide/ Agent	Concentration
									Dimethylcyclopropane carboxylic acid (DCCA; a metabolite of pyrethroid insecticides)	
				Urine Current child's concentrations		5-year study visit in 2015–2016			3,5,6-trichloro-2-pyridinol (TCPy; a metabolite of the OP chlorpyrifos)	30.85 µg/L
									Ethylenethiourea (ETU; a metabolite of mancozeb)	66.59 µg/L
									3-hydroxy-pyrimethanil (OHP; a metabolite of pyrimethan	445.67 µg/L

ID	Reference	Study group	Subjects	Sample	Cohort	Year	Place	Province	Pesticide/ Agent	Concentration
									il)	
									5-hydroxy-thiabendazole (OHT; a metabolite of thiabendazole)	20.26 μg/L
									2,4-dichlorophenoxyacetic acid (2,4-D; parent compound)	146.85 μg/L
									3-phenoxybenzoic acid (3-PBA; a metabolite of pyrethroid insecticides)	35.92 μg/L
									3-(2,2-dichlorovinyl)-2,2-Dimethylcyclopropane carboxylic acid	44.1 μg/L

ID	Reference	Study group	Subjects	Sample	Cohort	Year	Place	Province	Pesticide/ Agent	Concentration
									(DCCA; a metabolite of pyrethroid insecticides)	

Supplementary Table 4. Biomonitoring guidance values for the calculation of the HQ of each pesticide.

Pesticide	Sample	Concentration in Adults (µg/L)	Biomonitoring guidance value (ug/L)	HQ
Mancozeb	Urine	108.26 ±75.13	6000*	0.0180
	Blood	52.03 ± 2.85	57.91**	0.8985
Pyrimethanil	Urine	375.41 ± 298.59	6000*	0.0626
Thiabendazole	Urine	301.43 ± 161.56	6000*	0.0502
Chlorpyrifos	Urine	53.02 ± 30.44	2100	0.0252
2,4-D	Urine	58.18 ± 28.77	10500	0.0055
Glyphosate	Urine	2.55 ± 2.67	5400	0.0005
Propineb	Urine	5.67	6000*	0.0009
Paraquat	Urine	5.09 ± 3.59	6000*	0.0008
DDT	Blood	>0.05	57.91**	0.0009
Dieldrin	Blood	>0.25	57.91**	0.0043
Chlorothalonil	Blood	16.1	57.91**	0.2780
Lindane	Blood	>0.1	57.91**	0.0017
Tebuconazole	Urine	45.17	6000*	0.0075

*Average value of the BGV available for the other pesticides

**Average and converted value of the BGV available in blood for other pesticides

Supplementary Table 5. Values used for the calculation of the HQw of each pesticide.

Pesticide	Sample	Concentration in Adults (µg/L)	AOEL (mg kg^{-1} bw day^{-1})	Dermal penetration (%)	Absorbed permissible level (ug/L)	HQw
Mancozeb	Urine	108.26 ±75.13	0.011 (1)	0.11-0.24 (1)	9788.90	0.01106
	Blood	52.03 ± 2.85	0.011 (1)	0.11-0.24 (1)	3426.12	0.01519
Pyrimethanil	Urine	375.41 ± 298.59	0.120 (1)	1.0-46.0 (1)	14340102.86	0.00003
Thiabendazole	Urine	301.43 ± 161.56	0.070 (1)	0.15-1.84 (1)	354180.20	0.00085
Chlorpyrifos	Urine	53.02 ± 30.44	0.001 (2)	0.2-25 (1)	64072.80	0.00083
2,4-D	Urine	58.18 ± 28.77	0.020 (1)	0.1-4.0 (1)	208490.86	0.00028
Glyphosate	Urine	2.55 ± 2.67	0.100 (1)	1 (1)	508514.29	0.00001
Propineb	Urine	5.67	0.018 (1)	0.007 (1)	640.73	0.00885
Paraquat	Urine	5.09 ± 3.59	0.0004 (1)	0.5 (1)	1017.03	0.00500
DDT	Blood	>0.05	0.010 (3)	28 ± 2.9 (4)	498344.00	0.00000
Dieldrin	Blood	>0.25	0.0001 (5)	7.13-16.2 (6)	2076.14	0.00012
Chlorothalonil	Blood	16.1	0.003 (1)	0.02-1.2 (1)	3257.03	0.00494
Lindane	Blood	>0.1	0.001 (7)	9.72-10.78 (6)	18242.95	0.00001
Tebuconazole	Urine	45.17	0.030 (1)	13 (1)	1983205.71	0.00002

1. Lewis KA, Tzilivakis J, Warner DJ, Green A. An international database for pesticide risk assessments and management. Human and Ecological Risk Assessment: An International Journal. 2016;22(4):1050–64.
2. Authority EFS. Conclusion on the peer review of the pesticide human health risk assessment of the active substance chlorpyrifos. EFSA Journal. 2014;12(4):3640.
3. Wassie F, Spanoghe P, Tessema DA, Steurbaut W. Exposure and health risk assessment of applicators to DDT during indoor residual spraying in malaria vector control program. J Expo Sci Environ Epidemiol. 2012 Nov;22(6):549–58.
4. Moody RP, Nadeau B, Chu I. *In vitro* dermal absorption of pesticides: VI. *In vivo* and *in vitro* comparison of the organochlorine insecticide DDT in rat, guinea pig, pig, human and tissue-cultured skin. Toxicology in Vitro. 1994 Dec 1;8(6):1225–32.
5. Ministry of Health of New Zealand. Guidelines for drinking-water quality management for New Zealand [Internet]. 2019 [cited 2024 Nov 26]. Available from: https://view.officeapps.live.com/op/view.aspx?src=https%3A%2F%2Fwww.taumataarowai.govt.nz%2Fassets%2FUploads%2FMinistry-of-Health-drinking-water-datasheets%2Fdwg_vol3_datasheets_-_chemical_and_physical_determinands-part_2-3_pesticides.docx&wdOrigin=BROWSELINK
6. Fisher HL, Most B, Hall LL. Dermal absorption of pesticides calculated by deconvolution. Journal of Applied Toxicology. 1985;5(3):163–77.
7. Vergucht, S, Piñeros-Garcet, J, Pussemier, L, Steurbaut W. Development of a pesticide risk indicator for the evaluation of the Belgian reduction plan. In: Platform for scientific concertation: food safety [Internet]. Belgium: Belgiam Science Policy; 2007 [cited 2024 Nov 4]. p. 222–41. Available from: https://www.sciensano.be/sites/default/files/2007_berben_etal_2007_gmodetection.pdf#page=223

Supplementary Table 6. Pairwise comparison matrix for the criteria at level 2 of the hierarchy and the computed values of priority vector and consistency ratio.

	Criteria	Number of studies reported	Non cancerogenic risk	Endocrine Disruptor	Number of reproductive effects	Estimated use	Regulatory status International	Regulatory status Costa Rica	NOEC	Consistency ratio
Author 1	Number of studies reported	1	1/4	1/4	1/8	1	1/3	1/3	1/5	0.06
	Non cancerogenic risk	4	1	3	1/4	5	5	5	2	
	Endocrine Disruptor	4	1/3	1	1/5	3	3	3	1	
	Number of reproductive effects	8	4	5	1	8	5	7	2	
	Estimated use	1	1/5	1/3	1/8	1	1/3	1/2	1/5	
	Regulatory status International	3	1/5	1/3	1/5	3	1	3	1/5	
	Regulatory status Costa Rica	3	1/5	1/3	1/7	2	1/3	1	1/4	
	NOEC	5	1/2	1	1/2	5	5	4	1	

	Criteria	Number of studies reported	Non cancerogenic risk	Endocrine Disruptor	Number of reproductive effects	Estimated use	Regulatory status International	Regulatory status Costa Rica	NOEC	Consistency ratio
Author 2	**Number of studies reported**	1	1/9	1/9	1/8	1/7	1/6	1/6	1/8	0.03
	**Non cancerogenic risk**	9	1	1	2	3	4	4	2	
	**Endocrine Disruptor**	9	1	1	2	3	4	4	2	
	**Number of reproductive effects**	8	1/2	1/2	1	3	4	4	2	
	**Estimated use**	7	1/3	1/3	1/3	1	2	2	1/2	
	**Regulatory status International**	6	1/4	1/4	1/4	1/2	1	1	1/3	
	**Regulatory status Costa Rica**	6	1/4	1/4	1/4	1/2	1	1	1/3	
	**NOEC**	8	1/2	1/2	1/2	2	3	3	1	

	Criteria	Number of studies reported	Non cancerogenic risk	Endocrine Disruptor	Number of reproductive effects	Estimated use	Regulatory status International	Regulatory status Costa Rica	NOEC	Consistency ratio
Author 3	Number of studies reported	1	1/9	1/9	1/9	1/2	1/2	1/6	1/6	0.09
	Non cancerogenic risk	9	1	2	1/2	9	9	9	9	
	Endocrine Disruptor	9	1/2	1	1/5	7	7	7	3	
	Number of reproductive effects	9	2	5	1	8	8	8	3	
	Estimated use	2	1/9	1/7	1/8	1	1	1/2	1	
	Regulatory status International	2	1/9	1/7	1/8	1	1	1/4	1/6	
	Regulatory status Costa Rica	6	1/9	1/7	1/8	2	4	1	1/2	
	NOEC	6	1/9	1/3	1/3	1	6	2	1	

	Criteria	Number of studies reported	Non cancerogenic risk	Endocrine Disruptor	Number of reproductive effects	Estimated use	Regulatory status International	Regulatory status Costa Rica	NOEC	Consistency ratio
Author 4	Number of studies reported	1	2	1/2	1	2	2	2	4	0.08
	Non cancerogenic risk	1/2	1	1/2	2	2	2	2	2	
	Endocrine Disruptor	2	2	1	2	9	9	9	9	
	Number of reproductive effects	1	1/2	1/2	1	8	8	8	8	
	Estimated use	1/2	1/2	1/9	1/8	1	2	2	2	
	Regulatory status International	1/2	1/2	1/9	1/8	1/2	1	2	2	
	Regulatory status Costa Rica	1/2	1/2	1/9	1/8	1/2	1/2	1	2	
	NOEC	1/4	1/2	1/9	1/8	1/2	1/2	1/2	1	

	Criteria	Number of studies reported	Non cancerogenic risk	Endocrine Disruptor	Number of reproductive effects	Estimated use	Regulatory status International	Regulatory status Costa Rica	NOEC	Consistency ratio
Author 5	Number of studies reported	1	1/2	2	4	8	7	8	6	0.07
	Non cancerogenic risk	2	1	4	6	9	7	8	5	
	Endocrine Disruptor	1/2	1/4	1	3	7	5	6	4	
	Number of reproductive effects	1/4	1/6	1/3	1	6	4	5	3	
	Estimated use	1/8	1/9	1/7	1/6	1	1/4	1/2	1/5	
	Regulatory status International	1/7	1/7	1/5	1/4	4	1	3	1/3	
	Regulatory status Costa Rica	1/8	1/8	1/6	1/5	2	1/3	1	1/4	
	NOEC	1/6	1/5	1/4	1/3	5	3	4	1	

Supplementary Table 7. Pairwise comparison matrices of pesticides for each criterion with local priority vector and consistency ratios

**Number of studies in which it was reported**														
	Mancozeb	Pyrimethanil	Thiabendazole	Chlorpyrifos	2,4-D	Glyphosate	Propineb	Paraquat	DDT	Dieldrin	Chlorothalonil	Lindane	Tebuconazole	Priority vector
Mancozeb	1	3	5	2	4	9	9	9	9	9	9	9	9	0.27
Pyrimethanil	1/3	1	3	1/2	2	7	7	7	7	7	7	7	7	0.15
Thiabendazole	1/5	1/3	1	1/4	1/2	5	5	5	5	5	5	5	5	0.09
Chlorpyrifos	1/2	2	4	1	3	8	8	8	8	8	8	8	8	0.20
2,4-D	1/4	1/2	2	1/3	1	6	6	6	6	6	6	6	6	0.12
Glyphosate	1/9	1/7	1/5	1/8	1/6	1	1	1	1	1	1	1	1	0.02
Propineb	1/9	1/7	1/5	1/8	1/6	1	1	1	1	1	1	1	1	0.02
Paraquat	1/9	1/7	1/5	1/8	1/6	1	1	1	1	1	1	1	1	0.02
DDT	1/9	1/7	1/5	1/8	1/6	1	1	1	1	1	1	1	1	0.02
Dieldrin	1/9	1/7	1/5	1/8	1/6	1	1	1	1	1	1	1	1	0.02
Chlorothalonil	1/9	1/7	1/5	1/8	1/6	1	1	1	1	1	1	1	1	0.02
Lindane	1/9	1/7	1/5	1/8	1/6	1	1	1	1	1	1	1	1	0.02
Tebuconazole	1/9	1/7	1/5	1/8	1/6	1	1	1	1	1	1	1	1	0.02
Consistency ratio	0.0163													

Non-carcinogenic risk

	Mancozeb	Pyrimethanil	Thiabendazole	Chlorpyrifos	2,4-D	Glyphosate	Propineb	Paraquat	DDT	Dieldrin	Chlorothalonil	Lindane	Tebuconazole	Priority vector
Mancozeb	1	3	3	4	5	7	7	7	7	6	2	6	5	0.24
Pyrimethanil	1/3	1	1	2	3	5	5	5	5	4	1/2	4	3	0.12
Thiabendazole	1/3	1	1	2	3	5	5	5	5	4	1/2	4	3	0.12
Chlorpyrifos	1/4	1/2	1/2	1	2	4	4	4	4	3	1/3	3	2	0.08
2,4-D	1/5	1/3	1/3	1/2	1	3	3	3	3	2	1/4	2	1	0.05
Glyphosate	1/7	1/5	1/5	1/4	1/3	1	1	1	1	1/2	1/6	1/2	1/3	0.02
Propineb	1/7	1/5	1/5	1/4	1/3	1	1	1	1	1/2	1/6	1/2	1/3	0.02
Paraquat	1/7	1/5	1/5	1/4	1/3	1	1	1	1	1/2	1/6	1/2	1/3	0.02
DDT	1/7	1/5	1/5	1/4	1/3	1	1	1	1	1/2	1/6	1/2	1/3	0.02
Dieldrin	1/6	1/4	1/4	1/3	1/2	2	2	2	2	1	1/5	1	1/2	0.03
Chlorothalonil	1/2	2	2	3	4	6	6	6	6	5	1	5	4	0.17
Lindane	1/6	1/4	1/4	1/3	1/2	2	2	2	2	1	1/5	1	1/2	0.03
Tebuconazole	1/5	1/3	1/3	1/2	1	3	3	3	3	2	1/4	2	1	0.05
Consistency ratio	0.0173													

**Classification as an endocrine disruptor**														
	Mancozeb	Pyrimethanil	Thiabendazole	Chlorpyrifos	2,4-D	Glyphosate	Propineb	Paraquat	DDT	Dieldrin	Chlorothalonil	Lindane	Tebuconazole	Priority vector
Mancozeb	1	4	5	1	1	4	4	9	1	1	1	1	1	0.11
Pyrimethanil	1/4	1	2	1/4	1/4	1	1	6	1/4	1/4	1/4	1/4	1/4	0.03
Thiabendazole	1/5	1/2	1	1/5	1/5	1/2	1/2	5	1/5	1/5	1/5	1/5	1/5	0.02
Chlorpyrifos	1	4	5	1	1	4	4	9	1	1	1	1	1	0.11
2,4-D	1	4	5	1	1	4	4	9	1	1	1	1	1	0.11
Glyphosate	1/4	1	2	1/4	1/4	1	1	6	1/4	1/4	1/4	1/4	1/4	0.03
Propineb	1/4	1	2	1/4	1/4	1	1	6	1/4	1/4	1/4	1/4	1/4	0.03
Paraquat	1/9	1/6	1/5	1/9	1/9	1/6	1/6	1	1/9	1/9	1/9	1/9	1/9	0.01
DDT	1	4	5	1	1	4	4	9	1	1	1	1	1	0.11
Dieldrin	1	4	5	1	1	4	4	9	1	1	1	1	1	0.11
Chlorothalonil	1	4	5	1	1	4	4	9	1	1	1	1	1	0.11
Lindane	1	4	5	1	1	4	4	9	1	1	1	1	1	0.11
Tebuconazole	1	4	5	1	1	4	4	9	1	1	1	1	1	0.11
Consistency ratio	0.0125													

Number of negative effects reported on reproduction														
	Mancozeb	Pyrimethanil	Thiabendazole	Chlorpyrifos	2,4-D	Glyphosate	Propineb	Paraquat	DDT	Dieldrin	Chlorothalonil	Lindane	Tebuconazole	Priority vector
Mancozeb	1	2	2	2	1	1	2	2	1	1/4	1	1	2	0.08
Pyrimethanil	1/2	1	1	1	1/2	1/2	1	1	1/2	1/5	1/2	1/2	1	0.04
Thiabendazole	1/2	1	1	1	1/2	1/2	1	1	1/2	1/5	1/2	1/2	1	0.04
Chlorpyrifos	1/2	1	1	1	1/2	1/2	1	1	1/2	1/5	1/2	1/2	1	0.04
2,4-D	1	2	2	2	1	1	2	2	1	1/4	1	1	2	0.08
Glyphosate	1	2	2	2	1	1	2	2	1	1/4	1	1	2	0.08
Propineb	1/2	1	1	1	1/2	1/2	1	1	1/2	1/5	1/2	1/2	1	0.04
Paraquat	1/2	1	1	1	1/2	1/2	1	1	1/2	1/5	1/2	1/2	1	0.04
DDT	1	2	2	2	1	1	2	2	1	1/4	1	1	2	0.08
Dieldrin	4	5	5	5	4	4	5	5	4	1	4	4	5	0.26
Chlorothalonil	1	2	2	2	1	1	2	2	1	1/4	1	1	2	0.08
Lindane	1	2	2	2	1	1	2	2	1	1/4	1	1	2	0.08
Tebuconazole	1/2	1	1	1	1/2	1/2	1	1	1/2	1/5	1/2	1/2	1	0.04
Consistency ratio	0.0026													

Estimated use

	Mancozeb	Pyrimethanil	Thiabendazole	Chlorpyrifos	2,4-D	Glyphosate	Propineb	Paraquat	DDT	Dieldrin	Chlorothalonil	Lindane	Tebuconazole	Priority vector
Mancozeb	1	4	4	2	2	2	3	2	6	6	3	5	3	0.18
Pyrimethanil	1/4	1	1	1/3	1/3	1/3	1/2	1/3	3	3	1/2	2	1/2	0.04
Thiabendazole	1/4	1	1	1/3	1/3	1/3	1/2	1/3	3	3	1/2	2	1/2	0.04
Chlorpyrifos	1/2	3	3	1	1	1	2	1	5	5	2	4	2	0.12
2,4-D	1/2	3	3	1	1	1	2	1	5	5	2	4	2	0.12
Glyphosate	1/2	3	3	1	1	1	2	1	5	5	2	4	2	0.12
Propineb	1/3	2	2	1/2	1/2	1/2	1	1/2	4	4	1	3	1	0.07
Paraquat	1/2	3	3	1	1	1	2	1	5	5	2	4	2	0.12
DDT	1/6	1/3	1/3	1/5	1/5	1/5	1/4	1/5	1	1	1/4	1/2	1/4	0.02
Dieldrin	1/6	1/3	1/3	1/5	1/5	1/5	1/4	1/5	1	1	1/4	1/2	1/4	0.02
Chlorothalonil	1/3	2	2	1/2	1/2	1/2	1	1/2	4	4	1	3	1	0.07
Lindane	1/5	1/2	1/2	1/4	1/4	1/4	1/3	1/4	2	2	1/3	1	1/3	0.03
Tebuconazole	1/3	2	2	1/2	1/2	1/2	1	1/2	4	4	1	3	1	0.07
Consistency ratio	0.0111													

International Regulatory Status														
	Mancozeb	Pyrimethanil	Thiabendazole	Chlorpyrifos	2,4-D	Glyphosate	Propineb	Paraquat	DDT	Dieldrin	Chlorothalonil	Lindane	Tebuconazole	Priority vector
Mancozeb	1	5	4	1	3	3	1	1/2	1/5	1/5	1	1/5	4	0.05
Pyrimethanil	1/5	1	1/2	1/5	1/3	1/3	1/5	1/6	1/9	1/9	1/5	1/9	1/2	0.01
Thiabendazole	1/4	2	1	1/4	1/2	1/2	1/4	1/5	1/8	1/8	1/4	1/8	1	0.02
Chlorpyrifos	1	5	4	1	3	3	1	1/2	1/5	1/5	1	1/5	4	0.05
2,4-D	1/3	3	2	1/3	1	1	1/3	1/4	1/7	1/7	1/3	1/7	2	0.02
Glyphosate	1/3	3	2	1/3	1	1	1/3	1/4	1/7	1/7	1/3	1/7	2	0.02
Propineb	1	5	4	1	3	3	1	1/2	1/5	1/5	1	1/5	4	0.05
Paraquat	2	6	5	2	4	4	2	1	1/4	1/4	2	1/4	5	0.08
DDT	5	9	8	5	7	7	5	4	1	1	5	1	8	0.20
Dieldrin	5	9	8	5	7	7	5	4	1	1	5	1	8	0.20
Chlorothalonil	1	5	4	1	3	3	1	1/2	1/5	1/5	1	1/5	4	0.05
Lindane	5	9	8	5	7	7	5	4	1	1	5	1	8	0.20
Tebuconazole	1/4	2	1	1/4	1/2	1/2	1/4	1/5	1/8	1/8	1/4	1/8	1	0.02
Consistency ratio	0.0275													

Costa Rican Regulatory Status														
	Mancozeb	Pyrimethanil	Thiabendazole	Chlorpyrifos	2,4-D	Glyphosate	Propineb	Paraquat	DDT	Dieldrin	Chlorothalonil	Lindane	Tebuconazole	Priority vector
Mancozeb	1	1	1	1/5	1	1	1	1/5	1/9	1/9	1/9	1/9	1	0.02
Pyrimethanil	1	1	1	1/5	1	1	1	1/5	1/9	1/9	1/9	1/9	1	0.02
Thiabendazole	1	1	1	1/5	1	1	1	1/5	1/9	1/9	1/9	1/9	1	0.02
Chlorpyrifos	5	5	5	1	5	5	5	1	1/5	1/5	1/5	1/5	5	0.07
2,4-D	1	1	1	1/5	1	1	1	1/5	1/9	1/9	1/9	1/9	1	0.02
Glyphosate	1	1	1	1/5	1	1	1	1/5	1/9	1/9	1/9	1/9	1	0.02
Propineb	1	1	1	1/5	1	1	1	1/5	1/9	1/9	1/9	1/9	1	0.02
Paraquat	5	5	5	1	5	5	5	1	1/5	1/5	1/5	1/5	5	0.07
DDT	9	9	9	5	9	9	9	5	1	1	1	1	9	0.19
Dieldrin	9	9	9	5	9	9	9	5	1	1	1	1	9	0.19
Chlorothalonil	9	9	9	5	9	9	9	5	1	1	1	1	9	0.19
Lindane	9	9	9	5	9	9	9	5	1	1	1	1	9	0.19
Tebuconazole	1	1	1	1/5	1	1	1	1/5	1/9	1/9	1/9	1/9	1	0.02
Consistency ratio	0.0191													

NOEC (no-observed-effect concentration) - Chronic test in Daphnia magna (reproduction test)														
	Mancozeb	Pyrimethanil	Thiabendazole	Chlorpyrifos	2,4-D	Glyphosate	Propineb	Paraquat	DDT	Dieldrin	Chlorothalonil	Lindane	Tebuconazole	Priority vector
Mancozeb	1	4	4	1	7	7	4	4	5	5	1	7	4	0.19
Pyrimethanil	1/4	1	1	1/4	4	4	1	1	2	2	1/4	4	1	0.06
Thiabendazole	1/4	1	1	1/4	4	4	1	1	2	2	1/4	4	1	0.06
Chlorpyrifos	1	4	4	1	7	7	4	4	5	5	1	7	4	0.19
2,4-D	1/7	1/4	1/4	1/7	1	1	1/4	1/4	1/3	1/3	1/7	1	1/4	0.02
Glyphosate	1/7	1/4	1/4	1/7	1	1	1/4	1/4	1/3	1/3	1/7	1	1/4	0.02
Propineb	1/4	1	1	1/4	4	4	1	1	2	2	1/4	4	1	0.06
Paraquat	1/4	1	1	1/4	4	4	1	1	2	2	1/4	4	1	0.06
DDT	1/5	1/2	1/2	1/5	3	3	1/2	1/2	1	1	1/5	3	1/2	0.04
Dieldrin	1/5	1/2	1/2	1/5	3	3	1/2	1/2	1	1	1/5	3	1/2	0.04
Chlorothalonil	1	4	4	1	7	7	4	4	5	5	1	7	4	0.19
Lindane	1/7	1/4	1/4	1/7	1	1	1/4	1/4	1/3	1/3	1/7	1	1/4	0.02
Tebuconazole	1/4	1	1	1/4	4	4	1	1	2	2	1/4	4	1	0.06
Consistency ratio	0.0170													
